# Unveiling the Emerging Role of Xanthine Oxidase in Acute Pancreatitis: Beyond Reactive Oxygen Species

**DOI:** 10.3390/antiox14010095

**Published:** 2025-01-15

**Authors:** Chenxia Han, Yaling Wu, Juan Rong, Qing Xia, Dan Du

**Affiliations:** 1 West China Centre of Excellence for Pancreatitis, Institute of Integrated Traditional Chinese and Western Medicine, West China Hospital, Sichuan University, Chengdu 610041, China; 2 Frontiers Science Center for Disease-Related Molecular Network, West China Hospital, Sichuan University, Chengdu 610041, China; 3 Department of Gastroenterology, The Third People’s Hospital of Chengdu, Chengdu 610031, China

**Keywords:** xanthine oxidase, acute pancreatitis, drug target, cellular mechanism, multiple organ failure

## Abstract

Acute pancreatitis (AP) is a potentially fatal acute digestive disease that is widespread globally. Although significant progress has been made in the previous decade, the study of mechanisms and therapeutic strategies is still far from being completed. Xanthine oxidase (XO) is an enzyme that catalyzes hypoxanthine and xanthine to produce urate and is accompanied by the generation of reactive oxygen species (ROS) in purine catabolism. Considerable preclinical and clinical studies have been conducted over many decades to investigate the role of XO in the pathogenesis of AP and its potential targeting therapeutic value. There is no doubt that the ROS generated by irreversibly activated XO participates in the local pancreas and multiple organ failure during AP. However, the optimal timing and doses for therapeutic interventions targeting XO in animal studies and the clinic, as well as the additional molecular mechanisms through which XO contributes to disease onset and progression, including metabolic regulation, remain to be elucidated. This review summarized the benefits and contradictions of using XO inhibitors in animal models, offered mechanisms other than ROS, and discussed the difficulties faced in clinical trials. We hope to provide a perspective on the future worthwhile basic and clinical research on XO by analyzing its chemical and biological characteristics, as well as the progress of its regulatory mechanisms in AP.

## 1. The Major Challenge: Mechanism, Target, and Drug Discovery of Severe Acute Pancreatitis (SAP)

Acute pancreatitis (AP) is a prevalent gastrointestinal disorder marked by intense pain [1]. The incidence of AP has been increasing during recent decades [2]. Gallstones, alcohol, and triglyceridemia rank as the top etiologies [3]. Approximately 80% of AP patients are mild cases, which are mostly self-limited. But there remain 20% of AP patients who develop severe AP (SAP), with local or systemic complications [4]. The high mortality rate of SAP, ranging from 20% to 40%, has been regarded as a critical problem. Supportive treatments, including goal-directed fluid resuscitation, early oral feeding as tolerated, and enteral nutrition fluid control are conventional therapeutic approaches [5]. Nevertheless, evidence-based research [6] indicates that targeted therapy is still lacking, resulting in restricted clinical intervention for SAP [7]. Therefore, a key step in achieving therapeutic success is to hinder the transition from mild to SAP in the optimal treatment window.

By now, pathological research based on laboratory studies of AP has become the foundation of clinical efforts. Generally, the mechanisms can be categorized into two primary processes: disturbance of acinar cell homeostasis and the amplification of the inflammatory cascade [7]. The major pathological events in acinar cells that are responsible for the onset of AP include calcium overload, trypsinogen activation, mitochondrial dysfunction, and autophagy failure, etc. [8]. When local inflammatory damage progresses to systemic alterations, it will lead to the activation of multiple organ failure, primarily respiratory and circulatory failure, which in turn contributes to the high fatality rate in SAP [9]. Therefore, identifying the pathogenic processes and associated targets that contribute to and worsen the development of AP is the crucial step in achieving a substantial decrease in clinical mortality.

## 2. What Makes Xanthine Oxidase (XO) a Drug Target?

### 2.1. General Profile of XO and Target Characteristics

**Figure 1:**
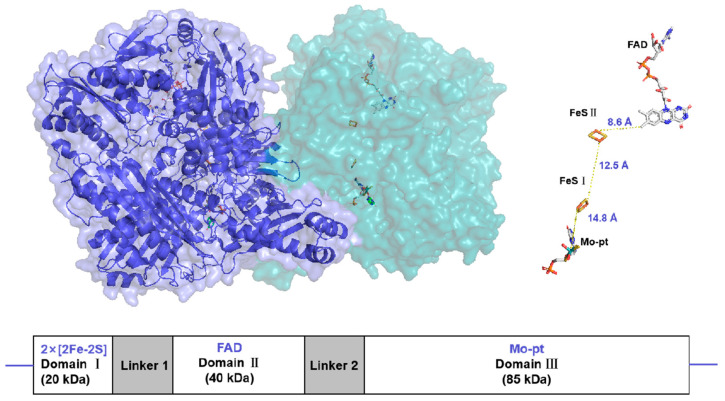
Surface representation of the Xanthine oxidoreductase (XOR) crystal structure homodimer (PDB:3NRZ). Monomer A is dark blue, while monomer B is light blue. The different protein cofactors (FAD, FeS II, FeS I, and Mo-pt), with the shortest distances between them marked, are represented on the right. The figure was created using PyMOL version 2.6.0.

Xanthine oxidoreductase (XOR) is a molybdenum-containing enzyme broadly distributed in biology [10]. The enzyme is a homodimeric protein with a net molecular weight of approximately 290 kDa and is composed of independent subunits. Each subunit contains two nonidentical [2Fe-2S]-type iron–sulfur clusters (Fe/S centers), an intermediate Flavin Adenine Dinucleotide (FAD) cofactor, and a molybdopterin (Mo-Pt) center [11] (Figure 1). The enzyme catalyzes hydroxylation of hypoxanthine to xanthine and xanthine to uric acid at the Mo-Pt center, and two electrons are thus rapidly transferred to FAD with the two Fe/S centers intervening, where an oxidative half-reaction occurs with concomitant reduction of nicotinamide adenine dinucleotide (NAD^+^) or molecular oxygen [12,13].

**Figure 2:**
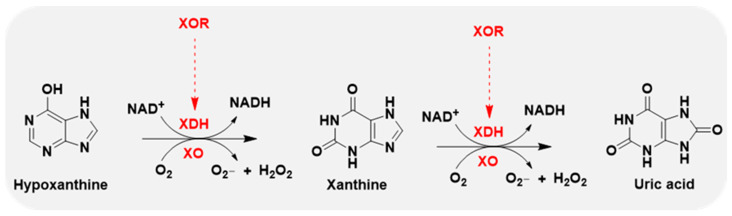
XOR continues to catalyze the oxidation of hypoxanthine and xanthine to uric acid, accompanied by the generation of reactive oxygen species (ROS), superoxide anion (O_2_^−^), and hydrogen peroxide (H_2_O_2_). XOR has 2 forms: xanthine dehydrogenase (XDH) and xanthine oxidase (XO). XDH prefers NAD^+^ as the substrate and XO prefers O_2_.

Xanthine oxidase (XO) is the oxidase form of XOR, and xanthine dehydrogenase (XDH) represents the dehydrogenase form of XOR. The oxidase and dehydrogenase forms are products of the same gene [14]. XDH shows a preference for NAD^+^ reduction at the FAD reaction site and generates NADH. In contrast, XO fails to react with NAD^+^ and exclusively uses dioxygen as its substrate, leading to the formation of reactive oxygen species (ROS), such as superoxide anion and hydrogen peroxide, which are major sources of ROS [15,16] (Figure 2).

**Figure 3:**
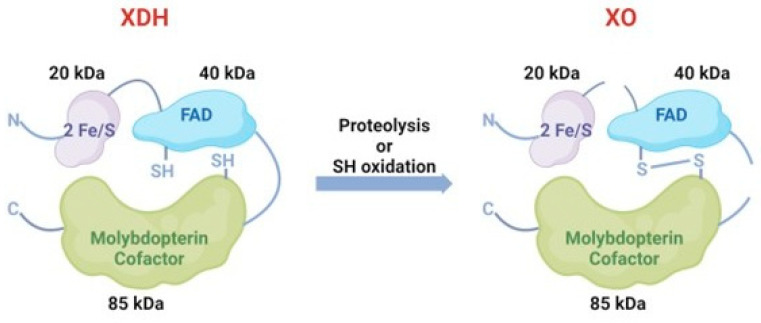
The conversion between XDH and XO. XDH could convert to XO via sulfhydryl side group oxidation reversibly or via proteolysis by serine proteases irreversibly.

They can convert to each other under different conditions. Generally, XDH may be converted to XO via sulfhydryl side group oxidation reversibly or via proteolysis by serine proteases irreversibly [17] (Figure 3). The former situation is usually induced by hypoxia conditions and the other transformation path is generally induced by prolonged ischemic conditions [18,19]. In pathological circumstances, hypoxia [20], trypsinogen activation leading to the irreversible conversion of XDH to XO [21] and the conversion of XDH to XO, is of major interest as it has been implicated in diseases characterized by oxygen radical-induced tissue damage, such as post-ischemic reperfusion injury [16].

XOR is present in all cell types, acting mainly as a dehydrogenase, and in most cases, with a low level of activity. The gene expression of XOR was found highest in the intestine and liver, with the highest enzyme activities correspondingly [22]; XOR is also detectable in other tissues, including the brain, heart, lung, kidney, skeletal muscle [23], and pancreas [24]. XOR is primarily produced by interferon in epithelial cells [25]; the leakage out of XOR may occur during various pathological conditions. Circulative XOR activity is associated with obesity, smoking, liver dysfunction, hyperuricemia, dyslipidemia, insulin resistance, adipokines [26], and AP [27]. Additionally, it has been reported that plasma XOR activity is much lower in humans than in animals [28].

### 2.2. Inhibitors of XO

The pathological and biological characteristics of XO render it a promising target for the development of XO inhibitors with therapeutic potential for various diseases. A multitude of different compounds have been discovered that can inhibit XO with varying degrees of selectivity and potency. According to their structures, XO inhibitors are often divided into purine-based and non-purine-based [29]. To date, clinically approved drugs based on XO inhibitors include allopurinol (**1**), febuxostat (**2**), and topiroxostat (**3**) [30]. Moreover, several synthetic and natural compounds with high selectivity for XO are summarized and presented (Table 1).

(1) Purine analogs

Allopurinol, the first US Food and Drug Administration (FDA)-approved purine-based XO inhibitor, has been widely used to treat gout for decades [50]. It has been extensively utilized in clinical practice due to its mechanism-based inhibition of uric acid synthesis; its active metabolite, oxypurinol, forms a covalent bond with the molybdenum ion within the XO enzyme, effectively curtailing uric acid production [51]. The clinical success of allopurinol has accelerated the development of a range of other purine-based XO inhibitors, including derivatives such as monosubstituted 2-(thioalkyl)purines [34] (**4**), 6-(N-benzoylamino)purine [35] (**5**), and 9-Benzoyl 9-deazaguanines [36] (**6**). Despite their therapeutic efficacy, the application of purine analogs is inherently limited by their structural similarity to endogenous purines. This similarity enables their conversion by hypoxanthine-guanine phosphoribosyltransferase and orotate phosphoribosyltransferase into nucleotide analogs, consequently disrupting normal purine metabolism and pyrimidine synthesis [52]. Such metabolic interference can lead to a series of adverse effects, including fever, allergic rash, diarrhea, leukopenia, nephrotoxicity, and liver damage [53]. Therefore, the broad therapeutic use of purine-based XO inhibitors has been limited, highlighting the need for novel, potent, non-purine XO inhibitors with fewer adverse effects.

(2) Non-purine analogs

- Synthetic non-purine analogs

The exploration of non-purine analogs has gained attention in the search for alternative XO inhibitors. Notable examples include febuxostat and topiroxostat, both of which have been extensively investigated for their inhibitory properties against XO. Febuxostat, a thiazole derivative, became one of the first non-purine XO inhibitors to receive FDA approval and acts as a highly potent non-competitive inhibitor of human XO [32]. It can fill the hydrophobic pocket, thereby inhibiting the activity of XO by obstructing substrate binding [54]. Topiroxostat, another significant non-purine XO inhibitor, was approved in Japan in 2013 [55]. It uniquely forms a covalent linkage with the molybdenum center of XO while simultaneously interacting with the enzyme’s hydrophobic pocket, thus categorizing it as a hybrid-type inhibitor [56]. However, both febuxostat and topiroxostat are associated with an increased risk of cardiovascular events and potential liver injury [57].

In light of these limitations, studies have been sought to develop novel molecules that exhibit both effectiveness and specificity in inhibiting XO. Innovations in the design of non-purine XO inhibitors have focused on exploiting the subpocket of the XO active site to create compounds with enhanced selectivity and diminished side effects. Various synthetic scaffolds have been introduced, including phenylthiazole-4-carboxylic acid derivatives, 1,2,3-triazole derivatives, aryl benzofuran derivatives, barbituric acid derivatives, pyrimidine derivatives, and beta-carboline alkaloid derivatives [52]. Notably, emerging candidates such as KUX-1151 and LC350189 have demonstrated significant potential as therapeutic interventions [30]. It is anticipated that successful clinical outcomes may render them viable drug candidates for patients with elevated uric acid levels, potentially leading to broader applications in the treatment of hyperuricemic conditions.

- Natural non-purine analogs

In addition to synthetic efforts, natural non-purine analogs have emerged as promising candidates for XO inhibition. Compounds such as flavonoids, chalcones, terpenoids, alkaloids, coumarins, phenolic acids, and phenylethanoid glycosides have exhibited significant inhibitory effects on XO activity [58]. Flavonoids, and in particular, natural phenolic compounds, have been documented to strongly inhibit XO in numerous studies. For instance, quercetin (**7**) stands out as a flavonoid that has advanced to clinical trials targeting hyperuricemia conditions; it has been shown to inhibit uric acid formation with an IC_50_ value of 2.79 ± 0.01 μM [37]. Structure–activity relationship studies suggest that flavonoids could bind to the active center of XO via hydrophobic interactions, prevent the entrance of substrate, and induce the rearrangement and conformation change of its secondary structures, leading to the inhibition of XO [59]. The planar configuration and specific C2 = C3 double bond in flavonoids promote binding and inhibitory activity, while modifications such as hydroxylation at ring B, C3 substitution, or methylation appear to diminish their potency [60]. Collectively, these findings underscore the necessity for further investigation into biocompatible XO inhibitors derived from natural products, aiming to identify low-toxicity alternatives with a favorable therapeutic profile.

## 3. XO Participates in AP Onset and Deterioration

### 3.1. XO and Its Generated ROS Are Related to the Etiology of AP

Damage to pancreatic acinar cells is the typical pathological feature of AP. Common AP risk factors, including fatty acids, gallstone, ischemia, and alcohol, can all induce the activation of XO, which results in massive ROS generation [61,62]. In sodium taurocholate (NaT)-induced AP, the XDH was fully transformed into XO within the initial 5 minutes, thereby generating oxygen free radicals (OFRs) [63]. This observation implies that XO may be one of the most important origins of OFRs that contribute to further damage of the pancreas [63]. Claus Niederau [64] observed that pancreatic acinar cells exposed to exogenous XO (20 mU/mL) induced iron-catalyzed (50 μM FeCl_3_) reactions on hypoxanthine (500 μM), leading to time-dependent damage. The main cytotoxic oxidant species produced in this process is H_2_O_2_. Hence, multiple etiologies can all trigger the process by which XDH is converted to XO and produces massive OFRs, suggesting a close relationship between XO and AP pathology [65]. However, although it has been recognized that OFRs can injure cells, deplete glutathione, and oxidate lipids and protein [66], the clinical treatments based on antioxidants in AP have not yet achieved success [6]. Other pathologies driven by XO activation gradually attract increasing attention [67].

### 3.2. XO Mainly Elevated in More Severe AP Animal Models

To investigate the alterations in XO activity in AP, Devenyi [68] initially quantified the XO activity of dehydrogenase and oxidase in pancreatic homogenates of mice. The present study documented that following three injections of cerulein at a dosage of 50 μg/kg, the activity of pancreatic XO remained in its dehydrogenase form rather than its oxidative form. The study proposed that the oxidative form of XO could be transformed by intrapancreatic protease and concluded that, for the first time, this conversion may take place in other models of AP rather than cerulein-induced mild AP mice model. This conclusion is supported by another study, which found that XO represents the main source of ROS in severe necrotic AP models [69], while the source of ROS in cerulein-induced edematous AP models is the infiltrated neutrophils [70].

The subsequent investigations demonstrated the obvious enhancement in XO in SAP models. The study conducted by D. Closa [69] revealed a notable increase in XO activity in rats with NaT-induced SAP in contrast to rats with cerulein-induced AP. Other studies also reported that in NaT-SAP models, XO appears to undergo substantial alterations and is favorably correlated with MPO levels [71]. Oxygen free radicals generated by xanthine and XO released to the bloodstream can directly lead to organ failure associated with AP [72]. Therefore, it looks like XO has a directly correlation with SAP pathogenesis.

Our recent study [67] revealed that in a model of cerulein-induced edematous AP, the expression of the *Xdh* gene in the pancreas showed a mild rise 8 hours after the initial injection. However, there was no significant increase in the XO protein level. The pancreatic mRNA level and serum enzyme activity of XO increased dramatically in an L-arginine-induced severe necrotic AP model within 8 hours of modeling and XO protein levels significantly increased at 72 hours. Furthermore, the severe AP model caused by NaT exhibited a notable rise in serum XO activity as early as 3 hours after modeling. Taken together, these data unequivocally indicated that XO was particularly upregulated in the SAP model rather than in mild examples.

### 3.3. Therapeutic Targeting of XO in Experimental AP

The classic XO inhibitors, allopurinol and its metabolite oxypurine, have been widely used in the treatments and prevention of AP (Table 2). According to the current literature, the effect of XO inhibitors on AP models seems controversial. However, this result is due to the time and dose of drug administration and, most importantly, the type of AP model. Thus, we summarized the experimental studies of the effect of XO inhibitors on AP according to the severity of AP models.

In necrotic SAP models, XO inhibitors including allopurinol, oxypurinol, and febuxostat all showed obvious beneficial effects on pancreatic injury and systematic inflammation. In the L-arginine-induced SAP models, both prophylactic and therapeutic usage of allopurinol with a dose range from 30 to 200 mg/kg were effective. In the case of the NaT-induced SAP model, one study from Lankisch, P.G. et al. [81] which conducted pretreatment of allopurinol with 100 mg/kg at 1 hour pre-modeling showed negative effects, while other therapeutic treatments all achieved protective effects with 20 or 200 mg/kg allopurinol or 5–10 mM oxypurinol [67,72,76,77,78,79,80]. Thus, in these SAP models, therapeutic treatments are recommended more than prophylactic administration. Regarding cerulein-induced mild AP, only one study from Wisner, J.R. et al. [87] found allopurinol with 20 mg/kg to be effective under therapeutic administration, while the other two prophylactic treatments did not achieve a beneficial effect [68,70]. The negative results in the cerulein model are reasonable because XO did not respond to cerulein stimulation [67,68]. Nevertheless, therapeutic administration still seems to be effective in this model, which needs more evidence to verify it. Other AP models also showed positive results when given both pre- and post-modeling, with doses ranging from 5 to 100 mg/kg [61,82,83,84,85,86], necessitating further investigation to assess dependability.

However, among the three XO inhibitors that have been experimentally used, the effective dose is distinct. For instance, the lowest effective dose is 30 mg/kg in L-arginine-induced SAP for allopurinol [67], 5 mM (approximately 6 mg/kg) for oxypurinol in NaT-induced SAP model [77,80], and 0.5 mg/kg for febuxostat in L-arginine SAP [67]. This result is consistent with their IC_50_ values, which are determined as 7.4 ± 0.07 μM [88] for allopurinol, 1.0 ± 0.5 μM for oxypurinol [89], and 1.8 nM for febuxostat [32]. The type of inhibitor also contributes to the variance among them. Allopurinol is a potent purine-like XO inhibitor, while febuxostat, a thiazole derivative, is a highly potent non-competitive inhibitor [90]. Oxypurinol is an active metabolite of allopurinol. Allopurinol can quickly oxidize to oxypurinol in vivo and functions as an irreversible covalent inhibitor [91].

Meanwhile, the timing for administration is another important factor impacting drug effects. In the case of the NaT-induced SAP mode, the drug dose can be totally different depending on the time for administration. For example, in the study of Isik, A.T. et al. [78], allopurinol was beneficial when it was given 2 hours after modeling with a high dose of 200 mg/kg. However, it has been found that the circulative XO activity was increased 3 h after modeling; accordingly, allopurinol administration 3 hours after modeling with a much lower dose of 20 mg/kg successfully achieved therapeutic effects [67]. Time for drug administration is also important for L-arginine-induced SAP. Although both allopurinol and febuxostat can improve the L-arginine-induced SAP model, it has been found that the administration time of allopurinol can be effective at 8 hours after modeling, while this timepoint can be delayed to 48 hours after modeling in the case of febuxostat. This result agrees with the fact that serum XO activity reached its peak at 48 hours after modeling [67]. In addition, the only positive effect of allopurinol in cerulein AP models is under therapeutic administration with continuous infusions, along with cerulein modeling [87]. It is worth noting that, in an ERCP-induced AP dog model, prophylactic treatment of allopurinol showed positive effects [83], which received inconsistent feedback in the following clinical research [31,92,93,94,95,96,97,98]. Thus, the administration time of XO inhibitors may depend on the changing pattern of local and systemic XO activity; it is safer and more effective when the drug is given during the peak period of local or circulative XO activation.

### 3.4. Cellular Mechanism of XO in Local Pancreatic Injury During AP

As a traditional target in AP, XO is recognized as an ROS resource in pancreatic acinar cells that activates inflammatory signals and oxidative stress-related cell death and effectively increases pancreatic enzymes [99]. A previous study revealed that the cellular mechanisms impacted by XO may be related to MAPK via inhibiting ERK 1/2 and JNK phosphorylation [77]. Additionally, these alterations were partially influenced by the calcium excess established by oxygen radicals [100]. In our recent research [67], we found that the inhibition of XO significantly protected against pancreatic acinar cell death by blocking the HIF-1α-NLRP3-signaling pathway. In the case of AP, hypoxia conditions are attributable to pancreatic necrosis. It has been reported that SAP patients generally suffered from deterioration of oxygen supply and consumption [101] and hyperbaric oxygen could ameliorate AP models [102]. Furthermore, hyperbaric oxygen combined with allopurinol could effectively improve AP [79]. Since the OFRs induced by XO activation can deplete oxygen and aggravate the hypoxia condition, the cellular pathology triggered by XO activation warrants further in-depth study.

**Figure 4:**
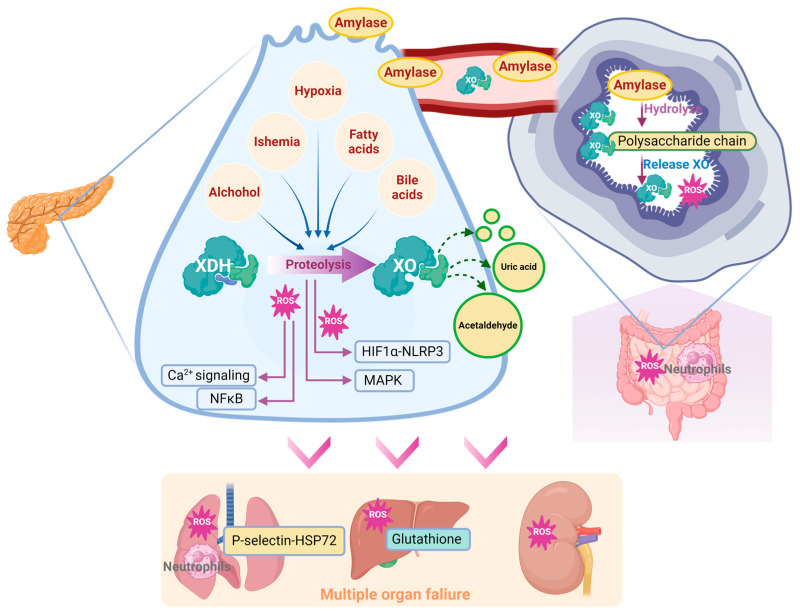
Schematic diagram of XO-mediated cellular mechanism during the onset and progression of AP.

The metabolic dysfunction that is triggered by XO activation also participates in AP progression. XOR is known as the rate-limiting enzyme in purine catabolism and is responsible for urate buildup. A significant increase in urate was found in the pancreata of SAP patients [67]. This finding is consistent with a recent prospective cohort study involving 124,316 people which found that the circulative urate level may be an important risk factor for AP [103]. Lactate and LDH are important molecules in cell death and SAP [104]. Purine catabolism was found to interfere with lactate metabolism in the pancreas of an AP mouse model and further metabolic flux analysis of pancreatic acinar cells suggested that XO inhibition can significantly restore lactate metabolism. Additionally, XO can catalyze other chemical reactions including the reversion from ethanol to acetaldehyde [62], which is an important cellular toxin during AP [105]. Collectively, XO-catalyzed metabolic dysfunction can directly or indirectly contribute to AP progression (Figure 4), which is worth investigating.

### 3.5. Involvement of XO in Distant Organ Failure During SAP

ROS are a crucial pathology related to the onset of multiple organ failure in SAP [106]. ROS play an important role in ischemia–reperfusion injury, sepsis, acute respiratory distress, and multiple organ dysfunction syndrome [107] by multiple mechanisms, including affecting the rate of apoptosis in tissue and endothelial cells of various organs [108]. Additionally, ROS immediately triggers ferroptosis via multiple mechanisms, like deubiquitinating and stabilizing *Sirt3* [109]. And it has been widely accepted that ferroptosis participates in AP [110] through exacerbating lipid peroxidation [111]. Notably, since XO is the major source of circulating ROS [112], it may have a significant effect on the systematic influences of SAP [27,65,113,114].

Lung is the most common organ that been attacked during AP progression. The oxygen in alveolar space is reported to be involved in XO-mediated lung inflammation [115]. XO combined with hypoxanthine intravenous infusion could significantly recruit polymorphonuclear leukocytes in the pancreas [116]. Correspondingly, inhibition of XO with 10 mM oxypurinol infusion significantly decreased SAP-associated lung injury and P-selectin, which functions as an inflammatory mediator inducing neutrophil infiltration [76,117]. Inhibition of XO also decreased NaT-induced lung heat shock protein 72 expression, which was activated by neutrophil infiltration in the early stage of SAP. These observations are in agreement with the conclusion that XO participates in neutrophil recruitment in AP [76]. In this case, XO could participate in lung injury during SAP via the lung neutrophil infiltration induced by XO and its generated ROS.

The significant impact of XO on SAP-induced lung injury may be related to its peripheral mobilization from the intestine. Under physiological conditions, XDH/XO is bound to the polysaccharide chains of heparin-like proteoglycans, which are located on the cell membrane of endothelial cells of the gastrointestinal system [27]. During AP, massive amylase has been released into the circulative system, due to its ability to hydrolyze the internal α-1,4 linkages of polysaccharides, which may disrupt the binding site of XDH/XOD and release XDH/XOD from the endothelial cell surface in the gastrointestinal system, indicating that amylase absorbed from ascitic fluid is involved in XO mobilization during AP [27]. Heparin could also aggravate AP-induced lung injury by inhibiting the binding between XO and endothelial cells [71].

**Figure 5:**
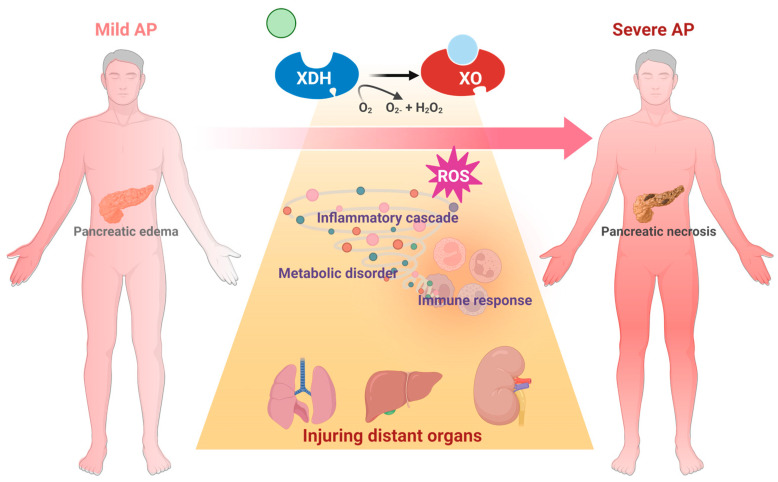
Schematic diagram of XO-mediated systemic mechanism from AP to SAP.

Other organs, including the liver and kidney, have also been associated with XO. Increased XO activity and reduced glutathione levels were found in the liver during NaT-induced SAP [72]. In the L-arginine-induced SAP model, XO inhibition with 200 mg/kg of allopurinol significantly decreased MDA activity in the pancreas and kidney [75]. Similarly, XO inhibition with 30 mg/kg of allopurinol also significantly improved liver and kidney dysfunction, evidenced by the reduction in serum urea, aspartate aminotransferase, and creatine levels in this model [67]. Interestingly, XO inhibition significantly decreased the oxidative stress in the kidney, but there seemed to be no change in the liver, which may be due to the better antioxidative ability of the liver [75]. Despite the current knowledge, the effect and mechanism of XO on other organs during SAP still need further research (Figure 5).

## 4. Clinical Trials of XO Inhibitors in AP Patients

In the clinic, the administration of XO inhibitors mainly focused on ERCP-induced AP patients (Table 3), which was based on the positive results of an AP canine model [83]. According to a meta-analysis, prophylactic use of allopurinol failed to prevent ERCP-induced AP [92,93]. Oral administration of 200, 300, or 600 mg of allopurinol 1–15 hours prior to ERCP exhibited a nonbeneficial effect on the incidence or severity of ERCP-AP [31,94,95,96]. On the contrary, a high dose of 600 mg of oral administration of allopurinol could protect against ERCP-induced AP, including promoting pancreatic injury and inhibiting the incidences of complications [97]. Another RCT using 300 mg also showed a protective effect against the incidences of hyperamylasemia–AP and high-risk progression [98]. Thus, the varied severity and etiology of AP, along with the dosage and timing of XO inhibitor administration, significantly influence the clinical outcomes of RCT. In addition, a retrospective review [118] claimed that among eight L-asparaginase-induced AP patients, three patients with oral administration of allopurinol of 120 mg/day for 8 days before pancreatitis, or 300 mg/day for 5 days before pancreatitis, or 200 mg/day for 5 days before pancreatitis and resumed after the resolution of pancreatitis for 14 days, all significantly improved in terms of computed tomography severity index and amylase level, compared with the patients who did not receive allopurinol. It cannot be concluded that if XO inhibition has beneficial effects on ERCP or other etiology-induced AP patients, then more RCTs are urgently needed in this field.

## 5. Limitations and Perspectives

Although multiple risk factors related to AP can activate XO and ROS generation, including fatty acids, ischemia, hypoxia, alcohol, and bile acids, the precise pathological mechanisms mediated by these triggers in the pancreas or multiple organs are unclear. It has been concluded that XO functions as an enhancer during the progression of SAP through ROS, hypoxia, catalytic ability, or metabolic regulation. However, the role of XO, a metabolic enzyme, in mediating metabolic imbalances and their effects on the pancreas and extra-pancreatic organs remains unclear. In this case, the communication mechanisms between organs and cells deserve further exploration. Due to the lack of genetic tools, the role of XO in different types of cells, such as immune cells, is unclear during AP. The dose and timing of XO inhibitor administration appear to be guided by changes in XO enzymatic activity; nevertheless, the comparative efficacy of different inhibitors and the accurate dose conversion between animal models and patients remain challenging to be determined. To provide scientific evidence for clinical applications, the dosage, treatment window, effectiveness, and safety of various inhibitors need to be deeply assessed and thoroughly compared. Regarding clinical research, the current studies merely focused on the effect of allopurinol on ERCP-induced AP, and probably failed to integrate how the significant enhancement in XO in severe cases and other etiologies induced AP. Future research welcomes the clinical use of a wider range of inhibitors, allowing for large-scale, multi-etiology, various severity, multi-center studies. Furthermore, it is hoped that clinical tests will incorporate indicators related to XO enzyme activity to diagnose SAP cases.

## Figures and Tables

**Table 1:** General chemical structural formulae of recently reported XO inhibitors.

Structure	Class	IC_50_ Value	Ref.
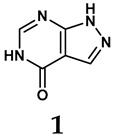	Purine derivatives	0.2–50 μM	[31]
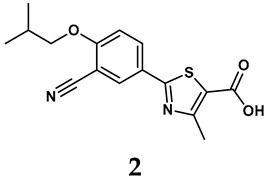	Thiazole derivative	0.0018 μM	[32]
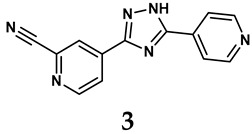	1,2,3-triazole derivative	0.0053 μM	[33]
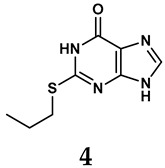	Purine derivatives	0.115 μM	[34]
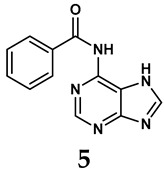	Purine derivatives	0.45 μM	[35]
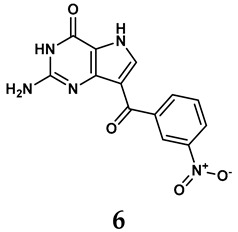	Purine derivatives	0.065 μM	[36]
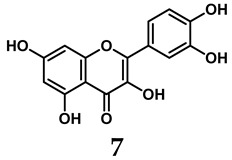	Flavonoid derivative	2.79 μM	[37]
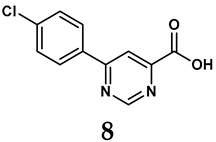	Pyrimidine derivative	18 μM	[38]
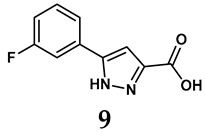	Pyrazole derivative	30 μM	[38]
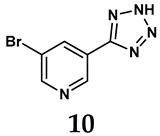	Isonicotinamide derivatives	64 μM	[38]
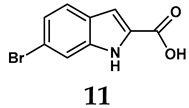	Imidazole derivatives	82 μM	[38]
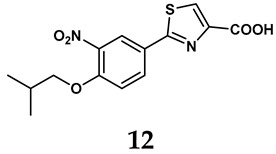	Thiazole derivative	0.0486 μM	[39]
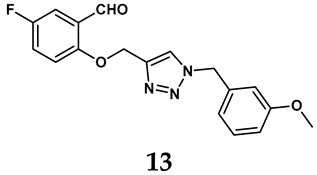	1,2,3-triazoles	0.70 μM	[40]
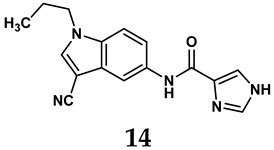	Imidazole derivatives	0.018 μM	[41]
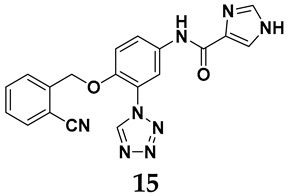	Isonicotinamide derivatives	0.022 μM	[42]
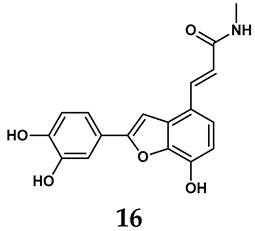	Benzo[*b*]furan derivatives	4.45 μM	[43]
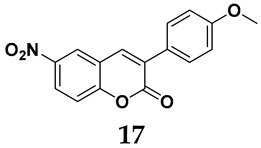	3-Phenylcoumarin derivatives	8.4 μM	[44]
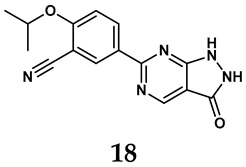	Pyrimidine derivatives	0.039 μM	[45]
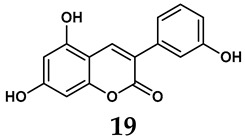	Coumarin analogs	2.13 μM	[46]
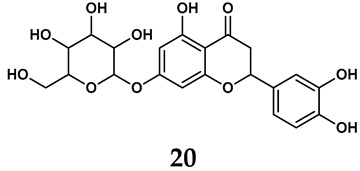	Flavonoid analogs	10.6 μM	[47]
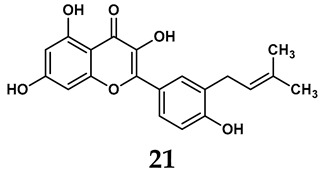	Flavonoid analogs	8.45 μM	[48]
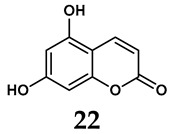	Coumarin analogs	10.91 μM	[48]
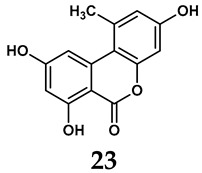	Ellagic acid analogs	0.23 μM	[49]

**Table 2:** The effects of XO inhibitors on AP experimental models.

Year	AP Model	Species	Drug	Dose	Administration	Effects	Drug Effect	Ref.
1998	L-arginine	Rat	Allopurinol	100 or 200 mg/kg	Prophylactic	↓Serum amylase; pancreatic MDA; histological damage; catalase activity ↑SOD; GPx	Positive	[73]
2000	L-arginine	Rat	Allopurinol	200 mg/kg	Prophylactic	↓Serum amylase; pancreatic MDA; histological damage	Positive	[74]
2000	L-arginine	Rat	Allopurinol	200 mg/kg	Prophylactic	↑Mn- and Cu; Zn-SOD GPx; catalase activity	Positive	[75]
2024	L-arginine	Mouse	Allopurinol and febuxostat	30 mg/kg for allopurinol and 0.5 mg/kg for febuxostat	Therapeutic	↓Serum amylase; histological damage; pancreatic trypsin activity; lung MPO ↑Serum SOD and GPx (only allopurinol)	Positive	[67]
	NaTC	Rat	Allopurinol	20 mg/kg		↓Serum amylase; histological damage ↑Serum SOD and GPx		
1998	NaTC	Rat	Oxypurinol	10 mM; 0.066 mL/min for 30 min	Therapeutic	↓Pancreatic MPO ↑GSH	Positive	[72]
1999	NaTC	Rat	Oxypurinol	10 mM; 0.066 mL/min for 30 min	Therapeutic	↓lung MPO	Positive	[76]
2004	NaTC	Rat	Oxypurinol	5 mM; 0.066 mL/min for 30 min	Therapeutic	↓Histological damage; lung MPO activity	Positive	[77]
2006	NaTC	Rat	Allopurinol	200 mg/kg	Therapeutic	↓Serum amylase; pancreatic MDA; histological damage ↑ Pancreatic SOD and GSH-Px	Positive	[78]
2007	NaTC	Rat	Allopurinol	200 mg/kg	Therapeutic	↓Serum amylase; pancreatic MDA; histological damage ↑Pancreatic SOD and GSH-Px	Positive	[79]
2012	NaTC	Rat	Oxypurinol	5 mM; 0.066 mL/min for 30 min	Therapeutic	↓Pancreatic and lung GSSG/GSH ratio; serum lipase	Positive	[80]
1989	NaTC	Rat	Allopurinol	100 mg/kg	Prophylactic	No change in serum amylase/lipase or pancreatic trypsin levels; no effect on histological damage, inflammation, and survival	Negative	[81]
	CDE	Mouse		50 mg/kg				
1985	CDE	Mouse	Allopurinol	24 mg/kg	Therapeutic	↓Serum amylase; pancreatic histological damage; survival rate	Positive	[82]
1998	ERCP	Dog	Allopurinol	5 mg/kg	Prophylactic	↓Serum amylase and lipase; histologic changes	Positive	[83]
2002	Ethyl alcohol	Dog	Allopurinol	10 mg/kg	Prophylactic	↓Pancreatic SOD	Positive	[84]
1985	FFA POSS ISCH	Dog	Allopurinol	1 mg/mL for 100 mL	Prophylactic	↓Serum amylase; pancreas edema; pancreatic weight	Positive	[61]
1991	ISCH + AA	Dog	Allopurinol	0.2 mg/mL for 500 mL	Therapeutic	↓Serum amylase; pancreas edema; pancreatic weight; hemorrhage	Positive	[85]
1992	PBDO + cerulein + ISCH	Rat	Allopurinol	20 mg/kg	Prophylactic	↓Serum amylase; histological changes	Positive	[86]
1987	Cerulein	Mouse	Allopurinol	0.7–7 mg/kg/h	Prophylactic	No effect on pancreas edema; no change in serum amylase level	Negative	[68]
1988	Cerulein	Rat	Allopurinol	20 mg/kg/h	Therapeutic	↓Serum amylase; pancreatic weight	Positive	[87]
1996	Cerulein	Rat	Allopurinol	50 mg/kg	Prophylactic	No change in serum amylase, lipase, and trypsin level; no effect on pancreatic histological damage	Negative	[70]

↓: Decrease; ↑: Increase; AA: acetaldehyde; CDE: choline-deficient ethionine-supplemented diet; ERCP: Endoscopic retrograde cholangiopancreatography; FFA: free fatty acid; GSH: glutathione; GSSG: oxidized glutathione; GSH-Px: glutathione peroxidase; ISCH: ischemia; MDA: Malondialdehyde; MPO: myeloperoxidase; NaTC: intraductal infusion of 5% sodium taurocholate; PBDO: pancreatico-biliary duct obstruction; POSS: partial duct obstruction combined with secretin stimulation; SOD: Superoxide dismutase.

**Table 3:** Randomized placebo-controlled trials of allopurinol for acute pancreatitis.

Year	Sample Size	Study Design	Intervention	Clinical Outcome	Ref.
2001	300	RCT	Allopurinol, 200 mg, 15 and 3 hours before ERCP vs. placebo	Neither prednisone nor allopurinol showed a beneficial influence on the incidence and severity of post-ERCP pancreatitis	[94]
2005	243	RCT	Allopurinol, 600 mg, 15 and 3 hours before ERCP vs. placebo	Pretreatment with a high dose of orally administered allopurinol decreased the frequency of post-ERCP pancreatitis	[97]
2005	701	RCT	Allopurinol, 600 and 300 mg, 4 and 1 hour before ERCP vs. placebo	Prophylactic oral allopurinol did not reduce the frequency or the severity of post-ERCP pancreatitis	[95]
2008	586	RCT	Allopurinol, 300 mg, 1 hour before ERCP vs. placebo	Allopurinol does not appear to reduce the overall risk of post-ERCP pancreatitis	[96]
2009	170	RCT	Allopurinol, 300 mg, 15 and 3 hours before ERCP vs. placebo	Oral allopurinol before ERCP decreased the incidences of hyperamylasemia and pancreatitis in patients submitted to high-risk procedures	[98]
2011	74	RCT	Allopurinol, 300 mg, 3 hours and just before ERCP vs. placebo	Allopurinol does not reduce the occurrence and amylase rise in post-ERCP pancreatitis	[31]

RCT: randomized controlled trial. ERCP: endoscopic retrograde cholangiopancreatography.
